# Supplementary material for: Impact of Human Papillomavirus Vaccination on Male Disease: A Systematic Review
Source: Vaccines (Basel). 2023 Jun 9;11(6):1083. doi: 10.3390/vaccines11061083 (PMC10302589; doi:10.3390/vaccines11061083)
Supplement: Supplementary file 1 [file vaccines-11-01083-s001.zip › vaccines-2410150-supplementary.pdf]

Supplementary Material S1

Impact of Human Papillomavirus Vaccination on Male Disease: A Systematic Review

Catarina Rosado [1], Ângela Rita Fernandes [1], Acácio Gonçalves Rodrigues [1,2] and Carmen Lisboa [1,2,3,*]

[1] Division of Microbiology, Department of Pathology, Faculty of Medicine, University of Porto, 4200-039 Porto, Portugal; up201708379@edu.med.up.pt (C.R.); arfernandes@med.up.pt (Â.R.F.); agr@med.up.pt (A.G.R.)
[2] CINTESIS@RISE, Center of Health Technology and Services Research/Rede de Investigação em Saúde, Faculty of Medicine, University of Porto, 4200-319 Porto, Portugal
[3] Department of Dermatology and Venereology, Centro Hospitalar Universitário São João, 4200-319 Porto, Portugal
* Correspondence: carlis@med.up.pt

Supplementary Material S1. Search strategy and study selection criteria

- *Search strategy*

Original search – 632 results, searched on 29/10/2022

Web Of Science – 183 results
(ALL=((human papillomavirus OR hpv) AND (vaccine OR vaccination OR immunization) AND (efficacy) AND (male disease OR males disease OR man disease OR men disease))) NOT DT=(Review) and 2010 or 2011 or 2012 or 2013 or 2014 or 2015 or 2016 or 2017 or 2018 or 2019 or 2020 or 2021 or 2022 (Publication Years)
Scopus - 273 results
(TITLE-ABS-KEY ((human AND papillomavirus OR hpv)) AND TITLE-ABS-KEY ((vaccine OR vaccination OR immunization)) AND TITLE-ABS-KEY ((efficacy)) AND TITLE-ABS-KEY ((male AND disease OR males AND disease OR man AND disease OR men AND disease))) AND (EXCLUDE (DOCTYPE , "re")) AND (LIMIT-TO (PUBYEAR , 2022) OR LIMIT-TO (PUBYEAR , 2021) OR LIMIT-TO (PUBYEAR , 2020) OR LIMIT-TO (PUBYEAR , 2019) OR LIMIT-TO (PUBYEAR , 2018) OR LIMIT-TO (PUBYEAR , 2017) OR LIMIT-TO (PUBYEAR , 2016) OR LIMIT-TO (PUBYEAR , 2015) OR LIMIT-TO (PUBYEAR , 2014) OR LIMIT-TO (PUBYEAR , 2013) OR LIMIT-TO (PUBYEAR , 2012) OR LIMIT-TO (PUBYEAR , 2011) OR LIMIT-TO (PUBYEAR , 2010))
MEDLINE (Pubmed) – 176 results
(human papillomavirus OR hpv) AND (vaccine OR vaccination OR immunization) AND (efficacy) AND (male disease OR males disease OR man disease OR men disease) NOT (review[Publication Type]) NOT (case reports[Publication Type]) Filters: from 2010 – 2022
(((("alphapapillomavirus"[MeSH Terms] OR "alphapapillomavirus"[All Fields] OR ("human"[All Fields] AND "papillomavirus"[All Fields]) OR "human papillomavirus"[All Fields] OR "hpv"[All Fields]) AND ("vaccin"[Supplementary Concept] OR "vaccin"[All Fields] OR "vaccination"[MeSH Terms] OR "vaccination"[All Fields] OR "vaccinable"[All Fields] OR

"vaccinal"[All Fields] OR "vaccinate"[All Fields] OR "vaccinated"[All Fields] OR "vaccinates"[All Fields] OR "vaccinating"[All Fields] OR "vaccinations"[All Fields] OR "vaccination s"[All Fields] OR "vaccinator"[All Fields] OR "vaccinators"[All Fields] OR "vaccine s"[All Fields] OR "vaccined"[All Fields] OR "vaccines"[MeSH Terms] OR "vaccines"[All Fields] OR "vaccine"[All Fields] OR "vaccins"[All Fields] OR ("vaccin"[Supplementary Concept] OR "vaccin"[All Fields] OR "vaccination"[MeSH Terms] OR "vaccination"[All Fields] OR "vaccinable"[All Fields] OR "vaccinal"[All Fields] OR "vaccinate"[All Fields] OR "vaccinated"[All Fields] OR "vaccinates"[All Fields] OR "vaccinating"[All Fields] OR "vaccinations"[All Fields] OR "vaccination s"[All Fields] OR "vaccinator"[All Fields] OR "vaccinators"[All Fields] OR "vaccine s"[All Fields] OR "vaccined"[All Fields] OR "vaccines"[MeSH Terms] OR "vaccines"[All Fields] OR "vaccine"[All Fields] OR "vaccins"[All Fields]) OR ("immune"[All Fields] OR "immuned"[All Fields] OR "immunes"[All Fields] OR "immunisation"[All Fields] OR "vaccination"[MeSH Terms] OR "vaccination"[All Fields] OR "immunization"[All Fields] OR "immunization"[MeSH Terms] OR "immunisations"[All Fields] OR "immunizations"[All Fields] OR "immunise"[All Fields] OR "immunised"[All Fields] OR "immuniser"[All Fields] OR "immunisers"[All Fields] OR "immunising"[All Fields] OR "immunities"[All Fields] OR "immunity"[MeSH Terms] OR "immunity"[All Fields] OR "immunization s"[All Fields] OR "immunize"[All Fields] OR "immunized"[All Fields] OR "immunizer"[All Fields] OR "immunizers"[All Fields] OR "immunizes"[All Fields] OR "immunizing"[All Fields])) AND ("efficacies"[All Fields] OR "efficacious"[All Fields] OR "efficaciously"[All Fields] OR "efficaciousness"[All Fields] OR "efficacy"[All Fields]) AND ((("male"[MeSH Terms] OR "male"[All Fields]) AND ("disease"[MeSH Terms] OR "disease"[All Fields] OR "diseases"[All Fields] OR "disease s"[All Fields] OR "diseased"[All Fields])) OR (("male"[MeSH Terms] OR "male"[All Fields] OR "males"[All Fields] OR "male s"[All Fields] OR "maleness"[All Fields]) AND ("disease"[MeSH Terms] OR "disease"[All Fields] OR "diseases"[All Fields] OR "disease s"[All Fields] OR "diseased"[All Fields])) OR (("men"[MeSH Terms] OR "men"[All Fields] OR "man"[All Fields]) AND ("disease"[MeSH Terms] OR "disease"[All Fields] OR "diseases"[All Fields] OR "disease s"[All Fields] OR "diseased"[All Fields])) OR (("men"[MeSH Terms] OR "men"[All Fields]) AND ("disease"[MeSH Terms] OR "disease"[All Fields] OR "diseases"[All Fields] OR "disease s"[All Fields] OR "diseased"[All Fields])))) NOT "review"[Publication Type]) NOT "case reports"[Publication Type]) AND (2010:2022[pdat])

<u>Additional search on 20/12/2022</u>

Clinical Trials.gov – 65 results
65 Studies found for: HPV Infection ǀ HPV vaccine ǀ Studies with Male Participants ǀ Start date from 01/01/2010 to 12/31/2022<br>Applied Filters: Male<br><br>Note: Condition or disease: HPV infection;<br>Intervention/treatment: HPV vaccine.

- *Study selection criteria*

**Inclusion criteria**	**Exclusion criteria**
Efficacy in males - presence or absence of clinical manifestations of HPV	Efficacy in females
All ages	Combined treatment (HPV vaccine plus other treatment)

Men with/without HPV infection at baseline	Cost-effectiveness studies
HPV vaccine as prevention of disease	Safety of the HPV vaccine
HPV vaccine as a treatment for a disease	Efficacy in males by female vaccination (herd effect)
Randomized controlled trials	Literacy about HPV and the HPV vaccine
Cohorts	Willingness to receive the HPV vaccine
Cross-sectional studies	Intralesional use of the HPV vaccine
To have a placebo or control group	Non-inferiority studies about the HPV vaccine doses
	Immunogenicity of the HPV vaccine
	Theoretical/Statistic models
	Studies about the prevalence of infection
	Studies about the incidence of infection
	Systematic Reviews
	Non-randomized controlled trials
	Reviews
	Case reports
	Animal studies

Supplementary Material S2

Impact of Human Papillomavirus Vaccination on Male Disease: A Systematic Review

Catarina Rosado [1], Ângela Rita Fernandes [1], Acácio Gonçalves Rodrigues [1,2] and Carmen Lisboa [1,2,3,*]

[1] Division of Microbiology, Department of Pathology, Faculty of Medicine, University of Porto, 4200-039 Porto, Portugal; up201708379@edu.med.up.pt (C.R.); arfernandes@med.up.pt (Â.R.F.); agr@med.up.pt (A.G.R.)
[2] CINTESIS@RISE, Center of Health Technology and Services Research/Rede de Investigação em Saúde, Faculty of Medicine, University of Porto, 4200-319 Porto, Portugal
[3] Department of Dermatology and Venereology, Centro Hospitalar Universitário São João, 4200-319 Porto, Portugal
* Correspondence: carlis@med.up.pt

Supplementary Material S2. Glossary of Nomenclature [30].

Anal HPV-related diseases:

<table>
<tr><td>High-grade anal intraepithelial neoplasia (HGAIN)</td><td rowspan="2">Anal intraepithelial neoplasia (AIN) grade 2|3</td></tr>
<tr><td>High squamous intraepithelial lesion (HSIL)</td></tr>
<tr><td>Low squamous intraepithelial lesion (LSIL)</td><td>Anal intraepithelial neoplasia (AIN) grade 1</td></tr>
</table>

Genital HPV-related diseases:

<table>
<tr><td>High-grade penile intraepithelial neoplasia (HGPIN)</td><td rowspan="2">Penile intraepithelial neoplasia (PIN) grade 2|3</td></tr>
<tr><td>High squamous intraepithelial lesion (HSIL)</td></tr>
<tr><td>Low squamous intraepithelial lesion (LSIL)</td><td>Penile intraepithelial neoplasia (PIN) grade 1</td></tr>
</table>
